# Patient with a Subarachnoid Headache

**DOI:** 10.5811/cpcem.2018.5.38417

**Published:** 2018-07-19

**Authors:** Ethan T. Montemayor, Brit Long, James A. Pfaff, Gregory P. Moore

**Affiliations:** * Boston University School of Medicine, Boston, Massachusetts; † Brooke Army Medical Center, Department of Emergency Medicine, San Antonio, Texas; ‡ Madigan Army Medical Center, Department of Emergency Medicine, Tacoma, Washington

## Abstract

Subarachnoid hemorrhage (SAH) is a life-threatening cause of headache. The diagnostic approach to this entity continues to evolve with a recent questioning of the classic workup of computed tomography and lumbar puncture. We report a risk management case of a patient with a missed SAH resulting in a fatal outcome. When there are multiple diagnostic strategies, the patient may be involved with shared decision-making. Some of the medical and legal implications of the diagnosis of SAH will be discussed.

## INTRODUCTION

Headaches are commonly seen in the emergency department (ED). Distinguishing a benign headache from a life- threatening one, such as subarachnoid hemorrhage (SAH), is a critical task for emergency physicians (EP). While the historical workup involves computed tomography (CT) followed by a lumbar puncture (LP), recent literature suggests that CT alone done within six hours obviates the need for this procedure. The following case will illustrate the significance in identifying the life-threatening diagnosis of SAH and the current state of the art in the diagnostic approach. We present a case, discuss the workup of SAH and the potential use of shared decision-making in this process.

## CASE

A 53-year-old male with a history of migraine headaches and sleep apnea was brought in by emergency medical services with the chief complaint of headache. He stated the headache had woken him from sleep approximately two hours prior to arrival, was in the occipital area, and was described as persistent, throbbing, sharp, and severe. He reported nausea, dizziness, trouble walking, and tingling of his extremities. He did not lose consciousness but described near syncope. The pain also was exacerbated by movement. He had a history of migraines; however, he stated this headache was different.

The review of systems was unremarkable. Physical exam revealed a blood pressure 134/87 millimeters of mercury, heart rate of 75 beats per minute, respiratory rate of 16 breaths per minute, oral temperature of 98.2º Fahrenheit, and oxygen saturation of 100%. He appeared mildly anxious and described an occipital headache, which was without meningismus and visual or neurological abnormalities. The remainder of the exam was unremarkable. The headache markedly improved with treatment. A noncontrast CT of the patient’s head was performed and interpreted as negative for masses or bleeds. A LP was performed with difficulty and revealed a large number of red blood cells (TNTC) but an absence of xanthochromia. Given the time frame, the difficulty with the procedure and the lack of xanthochromia, the providers interpreted this to be a traumatic LP. The EP prescribed metaclopromide, acetaminophen, decadron, promethazine and hydoromorphone with complete resolution of his symptoms. The patient was instructed to see his primary care physician for follow-up care.

The patient was seen in follow-up four days later in an outpatient setting. His labs were reviewed, and it was arranged for him to follow up with a neurologist. He was found dead at home the next day with a SAH secondary to a saccular aneurysm involving the anterior cerebral artery. In retrospect, the family stated that he had developed a headache the evening before his ED visit while weightlifting.

## DISCUSSION

### Montemayor

Headache accounts for approximately 2% of ED visits, with SAH occurring in 0.5% to 6%.^1^ Approximately 15% of patients with SAH will die before they reach the hospital, 25% die within 24 hours, and 45% of patients die within 30 days.^2^ Most patients with SAH experience abrupt headache, often thunderclap in nature, that reaches maximal intensity within one minute.^3^ Unfortunately, approximately 53% of cases are missed on initial presentation.^2^ Consequently, providers should have a low threshold of suspicion for SAH when patients present with key historical features. Such symptoms include sudden onset, difference in severity or quality compared to previous headaches and other symptoms, particularly neck stiffness, but also seizure, syncope, focal neurological deficit, and vomiting.^2^ Clinicians should consider that thunderclap headache is not specific for SAH. (15% of thunderclap headaches are the result of SAH.)^2^

Sentinel headaches are similar to SAH headaches, which may occur days to weeks prior to aneurysm rupture. The incidence appears in 10%-43% of patients with subsequent aneurysmal SAH.^4^ While certain signs and symptoms may increase or decrease the likelihood of SAH, no single characteristic of the history or physical exam is sufficient to rule in or rule out SAH.^5^

### Long

Noncontrast CT is done in the initial workup of a SAH. While older generation CT scanners had sensitivities approaching 92%, current generation machines demonstrate sensitivities approaching 100% if completed within six hours of headache onset.^6^ Several studies, both prospective and retrospective, have evaluated patients with sudden onset of headache, use of higher generation CT within six hours of onset, and CT interpreted by an experienced radiologist.^7^,^8^ The sensitivity and specificity approached 100%, though this is potentially limited in patients with anemia, smaller hemorrhage volume, poor CT quality, experience level of the interpreting radiologist, and imaging artifacts.^2^ This has generated a great deal of discussion with some authorities recommending this approach without performing a LP.^9^,^10^

Since many EPs are changing their practice based on these studies, it is imperative that the patient history is accurate. Despite little evidence for it, revisiting the history after the patient’s pain improves or soliciting family member may improve the history’s accuracy. In a patient with a sudden, severe headache with a normal neurologic examination, literature support exists for a negative noncontrast CT read by a qualified radiologist placing the patient at less than 1% risk for SAH.^6^,^11^

### Pfaff

While LP is classically used in the case of a negative CT and is a common procedure, it can result in a number of complications including post-spinal headache or back pain, infection, and spinal hematoma. Traumatic taps may occur in 15% of LPs, though the true frequency may be unknown and depends on how a traumatic tap is defined.^12^ LP can also provide an alternative diagnosis such as meningitis 3% of the time,^13^ but may not be beneficial if the pretest probability for SAH is low.^5^

Although all values in the cerebral spinal fluid analysis should be evaluated, red blood cells and xanthochromia are most commonly used to diagnosis SAH. Clearing of blood from successive tubes is unreliable since it can also occur with SAH.^14^ Classically, a decrease of red blood cells from the first to the fourth tubes has been used, but it is rare to completely clear. There is no clear cell count consensus with lower cutoffs anywhere from 100 × 10^6^ to 2,000 × 10^6^ cells per high power field or greater.^15^,^16^ Xanthochromia, a byproduct of hemoglobin breakdown, generally takes anywhere from 2–12 hours to develop. It may be measured by either spectrophotometry or visual inspections. Most laboratories use visual inspection performed by technicians. Studies have shown a wide variation in sensitivity of visual inspection.^6^ Xanthochromia in the setting of SAH greatly reduces the likelihood of a traumatic tap.^2^ This may not be helpful if a tap was done in the first six hours since only 20% of patients getting an LP during that time frame will have positive xanthochromia.^2^

### Pfaff/Moore

Failure to diagnose is the most common reason EPs are involved in litigation. Failing to accurately interpret a test or varying from accepted practice could put providers at risk for litigation. In the headache patient presenting within the first six hours, many EPs are using head CT results alone vs. the traditional practice of CT followed by LP and its potential complications. Since there is a low risk of missed SAH in patients with CT alone in the first six hours, these patients are good candidates for shared decision-making. Shared decision- making is the process of clinician and patient jointly participating in a healthcare decision after discussing the options, benefits, and harms, and considering the patient’s values, preferences, and circumstances.^17^,^18^ Prior to the discussion, the patient must have the capacity to understand the risks; there should be clear documentation of the information provided, with the understanding that it is the patient’s decision. Other factors include situations where there is more than one clinical option and equipoise, the patient has decision-making capability, and there is sufficient time for the physician and patient to make an informed decision.^19^ If adequate shared decision-making is done and adequately documented, the physician will likely have established a defense in the event the patient were to bring suit for malpractice.

### Moore

This legal concept and defense is called assumption of risk. In this situation, the patient is made aware of the risk and danger but makes the choice anyway. The legal elements of the assumption-of-risk defense are as follows: 1) The risk is known; 2) the risk is appreciated; and 3) the risk is voluntarily ignored. An early example of this was in *Charrin v Methodist Hospital, a* case in which a patient pointed out a television cord running across a room and later tripped over it. Her lawsuit was unsuccessful since she was aware of the risk of the cord.^20^ It is imperative that the risks are clearly spelled out. A classic legal case is *Schneider v Revici;* in this case a physician who practiced unorthodox treatment of breast cancer advised his patient to evaluate conventional treatment and then had her sign a consent form for treatments not adopted by the medical community (i.e., not standard of care). She later sued, and the appeals court acknowledged the assumption-of-risk defense and allowed the physician to use it.^21^

## CONCLUSION

While the approach to evaluation of the acute onset of headache evolves, it is imperative that the right patient population is chosen and that the history is accurate. When shared decision-making is used, the patient must have capacity, should understand the risk, and be informed that it is their decision. If this approach is used, the provider will very possibly have the availability of the assumption-of-risk defense in the event of adverse outcome.

## TAKE HOME POINTS

1. Knowledge of the full spectrum of presentations of SAH is important, as patients do not always present with the classic thunderclap headache.
2. CT is the imaging modality of choice if SAH is suspected. Further evaluation for SAH should be based on consideration of other diseases, time of headache onset, and other factors.
3. Ensure there is an accurate history when deciding to use the CT as the sole diagnostic tool in patients who present within six hours of onset, if an LP is not performed.
4. It is important for shared decision-making to have clear documentation of the information provided, the patient’s capacity, and the patient’s decision.
5. If shared decision-making is done, then it is possible for a physician to use the assumption-of-risk defense.

Documented patient informed consent and/or Institutional Review Board approval has been obtained and filed for publication of this case report.
